# “I will leave the baby with my mother”: Long‐distance travel and follow‐up care among HIV‐positive pregnant and postpartum women in South Africa

**DOI:** 10.1002/jia2.25121

**Published:** 2018-07-19

**Authors:** Kate Clouse, Matthew P Fox, Constance Mongwenyana, Molebogeng Motlhatlhedi, Sizakele Buthelezi, Dorah Bokaba, Shane A Norris, Jean Bassett, Mark N Lurie, David M Aronoff, Sten H Vermund

**Affiliations:** ^1^ Vanderbilt Institute for Global Health Vanderbilt University Nashville TN USA; ^2^ Division of Infectious Diseases Department of Medicine Vanderbilt University Nashville TN USA; ^3^ Department of Internal Medicine Health Economics and Epidemiology Research Office (HE^2^RO) School of Clinical Medicine Faculty of Health Sciences University of the Witwatersrand Johannesburg South Africa; ^4^ Department of Global Health Boston University School of Public Health Boston University Boston MA USA; ^5^ Department of Epidemiology Boston University School of Public Health Boston University Boston MA USA; ^6^ MRC/Wits Developmental Pathways for Health Research Unit University of the Witwatersrand Johannesburg South Africa; ^7^ Witkoppen Health and Welfare Centre Johannesburg South Africa; ^8^ Hikhensile Clinic City of Johannesburg Johannesburg South Africa; ^9^ Department of Epidemiology Brown University School of Public Health Providence RI USA; ^10^ Department of Epidemiology of Microbial Diseases and Center for Interdisciplinary Research on AIDS Yale University School of Public Health New Haven CT USA

**Keywords:** HIV/AIDS, mobility, pregnant, postpartum, retention, South Africa

## Abstract

**Introduction:**

It is common in urban African settings for postpartum women to temporarily return to family in distant settings. We sought to explore mobility among peripartum HIV‐positive women to understand the timing and motivation of travel, particularly vis‐à‐vis delivery, and how it may affect healthcare access.

**Methods:**

Using the same mobility measurements within three different studies, we examined long‐distance travel of mother and infant before and after delivery in three diverse clinics within greater Johannesburg, South Africa (n = 150). Participants were interviewed prior to delivery at two sites (n = 125) and after delivery at one (n = 25). Quantitative and qualitative results are reported.

**Results:**

Among 150 women, median age was 29 years (IQR: 26 to 34) and 36.3% were employed. Overall, 76.7% of the participants were born in South Africa: 32.7% in Gauteng Province (Johannesburg area) and 44.0% in other South African provinces, but birthplace varied greatly by site. Almost half (44.0%) planned to travel around delivery; nearly all after delivery. Median duration of stay was 30 days (IQR: 24 to 90) overall, but varied from 60 days at two sites to just 7 days at another. Participants discussed travel to eight of South Africa's nine provinces and four countries. Travel most frequently was to visit family, typically to receive help with the new baby. Nearly all the employed participants planned to return to work in Johannesburg after delivery, sometimes leaving the infant in the care of family outside of Johannesburg. All expressed their intent to continue HIV care for themselves and their infant, but few planned to seek care at the destination site, and care for the infant was emphasized over care for the mother.

**Conclusions:**

We identified frequent travel in the peripartum period with substantial differences in travel patterns by site. Participants more frequently discussed seeking care for the infant than for themselves. HIV‐exposed children often were left in the care of family members in distant areas. Our results show the frequent mobility of women and infants in the peripartum period. This underscores the challenge of ensuring a continuity of HIV care in a fragmented healthcare system that is not adapted for a mobile population.

## 1 Introduction

South Africa is home to the world's largest public antiretroviral therapy (ART) programme 1. This ambitious treatment programme has brought about historic gains in life expectancy 2, but is challenged by sub‐optimal retention in care 3, 4. The recent policy shift to a universal test‐and‐treat strategy 5 will increase the numbers of individuals on treatment but requires innovative approaches to initiate and retain all its estimated 7 million HIV‐infected adults and children on lifelong ART 6.

To address this high rate of attrition, pregnant, HIV‐positive women will require special attention, as they have been shown to be at high risk of disengagement from HIV care, particularly after delivery 7, 8, 9, 10, 11. In a Johannesburg cohort of women diagnosed with HIV during antenatal care, just under half were lost to HIV care within six months of delivery 10; a Cape Town study found that disengagement was more than twice as frequent in the postpartum period than during antenatal care 11. South African guidelines recommend five antenatal visits, two postpartum visits for the mother and child together, and then a return to routine ART care for HIV‐positive women 12. Retention through these multiple steps is a known challenge 13, 14.

One important reason that postpartum women have such high rates of attrition relates to mobility. Postpartum women are highly mobile for a number of reasons. South Africa has a long history of temporary labor migration as a legacy of apartheid 15, 16. Population mobility remains high today in democratic South Africa, and the most typical pattern is of internal migrants moving within South Africa from rural or peri‐urban areas to urban, but keeping strong ties to the origin 15. Migrants in general are able to return home much more frequently than in the past 17. Women migrate frequently too, are more likely than men to send remittances home, and may turn to family and neighbourhood networks to support children in their absence 16, 17. Postpartum women in South Africa often return to a rural home after delivery to receive care from family member 8, 17, 18, but the geospatial elements of this mobility are poorly characterized and their impact on retention in HIV care is unknown**.** The specific details of postpartum short‐term migration that could illuminate health services planning include the reasons for travel, the timing of departure in relation to delivery, the duration of this stay, and any attempts or obstacles to continuing care in the rural location or at the original clinic.

South Africa's health facilities are not linked through electronic medical records, so it is difficult to ascertain if a patient who is lost from one clinic seeks care at another 19. We recently investigated continued HIV care among women considered lost to follow‐up after initiating ART during pregnancy and found evidence that over one‐third had continued their HIV care at other clinics, both within the same city and throughout the country 20. The goal of the current study was to characterize mobility among peripartum (pregnant and postpartum) HIV‐positive women to understand the timing and motivation of travel, particularly vis‐à‐vis delivery, and how it may affect women's healthcare access.

## 2 Methods

Data were collected by nesting the same mobility‐related questions in data collection tools of three separate studies with diverse study objectives. Table S1 provides additional information on each study; all studies were related to understanding and improving engagement in HIV care among peripartum women. We enrolled 150 adult (age ≥ 18 years) peripartum participants at three study sites in Johannesburg, South Africa; activities and participant eligibility varied slightly at each site according to the objectives of each parent study.

Site one is a public health clinic operated by the City of Johannesburg that serves the Ivory Park region of eastern Johannesburg. Care is provided free of charge 21. Site one participants were recruited during routine antenatal care if they were pregnant, HIV positive, and able to speak and understand English. From May 2015 to March 2016, we enrolled 100 participants and conducted a one‐time questionnaire at enrolment.

Site two is a large primary healthcare clinic operated by a non‐governmental organization based in Fourways in northern Johannesburg; clinic visits cost R110 (USD~8.50), but fees may be waived if clients are unable to pay. Site two participants were recruited during routine antenatal care and eligible for enrolment if pregnant and HIV positive; we enrolled 25 participants from October 2016 to April 2017.

Site three is an academic research clinic located within Chris Hani Baragwanath Hospital in Soweto, on the southwestern edge of greater Johannesburg. Participants were already enrolled in a longitudinal maternal health cohort. Study visits are free and participants receive R150 (USD~11.60) for every completed visit. Women at site three were eligible for the present study if they were postpartum (gave birth 6 to 18 months prior to enrolment), HIV positive, and exhibiting a metabolic disorder (e.g. gestational diabetes). Interviews were conducted from August to December 2016. At sites two and three, we conducted a one‐time, in‐depth interview at enrolment based on a semi‐structured questionnaire guide. All interviews were conducted by a female trained research coordinator in the local language preference of the participant.

The residential areas served by site one and two's patient populations were developed in the 1990s 22, 23; both are densely‐populated areas with vast formal and informal settlements. By comparison, site three serves the population of a township that was formally established in 1963 24. The three diverse urban sites and mix of postpartum HIV‐infected women enabled us to examine migration patterns for diverse clients of a government clinic, a clinic run by an NGO, and a clinic run by an academic medical centre that aided women with metabolic disorders.

Study questions are presented in Table S2. At all sites, participants were asked similar initial yes/no questions regarding travel outside of the Johannesburg area before and after delivery. For participants at sites one and two, we asked about intended travel, while we asked about actual travel for the postpartum women enrolled at site three. If travel was noted, we recorded details of the duration, reason, and plans for travel. The questionnaire used at site one collected categorical, short answer, and some open‐ended responses. At sites two and three, in‐depth interviews explored experiences of travel and pregnancy. At site one, questionnaire data were captured on paper forms, then entered into a REDCap (Research Electronic Data Capture) electronic database 25. At sites two and three, interviews were recorded and transcribed.

When reporting the timing of travel, travel that was indicated to begin during pregnancy and end after delivery was marked as travel both before and after delivery. For example, if a pregnant participant reported planning to travel prior to delivery and staying through three months post‐delivery, both “before delivery” and “after delivery” travel would be noted for the same participant. All participants provided written informed consent prior to interviewing, and study activities were approved by the institutional review board of Vanderbilt University Medical Center, Boston University (site one), and the Human Research Ethics Committee of the University of the Witwatersrand.

### 2.1 Data analysis

SAS^®^ 9.4 (SAS Institute, Cary, NC, USA) was used for statistical analysis of quantitative data. Cohort characteristics are described using counts and proportions for categorical variables, and medians and interquartile ranges (IQR) for continuous data. Mobility within and outside of South Africa was mapped using ArcMap^®^ 10.3.1 (Esri, Inc., Redlands, CA, USA).

For the analysis of open‐ended data, coding, analysis, and reporting was completed by following the COREQ guidelines 26. Responses to questions related to mobility during the peripartum period (see list Table S2) were consolidated in REDCap and exported for hand‐coded analysis. Quotes were sorted by category, frequency distributions were examined, then quotes were read in detail to identify higher‐order themes and relationships. The analysis was rooted in the theoretical framework proposed by Phillips and Myer 27, which is an adaptation of the Social‐Ecological Model 28, and asserts that multi‐level factors interact to determine engagement in HIV care among pregnant and postpartum women. We report the themes identified – staying with family, childcare and children separated from the mother, and plans for continuing care – and highlight key, illustrative quotes.

## 3 Results

### 3.1 Participant characteristics

Participant characteristics are summarized in Table 1. Overall median age at enrolment was 29 years (IQR: 26 to 34), but varied across site, with the youngest participants at site one (median 28, IQR: 24 to 31), and the oldest at site three (median 36, IQR: 32 to 40). Median gestational age at enrolment was 26.7 weeks (IQR: 18.8 to 33.1); among the postpartum women at site three, the median time between delivery and interview was 10.7 months (IQR: 8.2 to 13.9). At site one, 28.0% of the participants were pregnant for the first time; at site two, 8.0% had never been pregnant before the current pregnancy. At both sites, among the majority who already had children, the median number of children was two (IQR: 1 to 2). Data on previous children were unavailable at site three. All participants were on lifelong, combination ART.

**Table 1:** Characteristics of the study participants at time of study enrolment (n = 150)

Participant characteristics	Site one (n = 100)	Site two (n = 25)	Site three (n = 25)	Total
Age in years, *median (IQR)*	28 (24 to 31)	33 (26 to 34)	36 (32 to 40)	29 (26 to 34)
Age, *n (%)*				
18 to 24 years	26 (26.0%)	4 (16.0%)	1 (4.0%)	31 (20.7%)
25 to 34 years	61 (61.0%)	16 (64.0%)	10 (40.0%)	87 (58.0%)
35 years and older	13 (13.0%)	5 (20.0%)	14 (56.0%)	32 (21.3%)
Current pregnancy is first pregnancy	28 (28.0%)	2 (8.0%)	Unavailable	30 (24.0%)^a^
Number of living children among those with 1+ children*, median (IQR)*	2 (1 to 2)	2 (1 to 2)	Unavailable	2 (1 to 2)
Participant reports a current partner, *n (%)*	99 (99.0%)	24 (96.0%)^b^	22 (88.0%)^c^	145 (96.7%)
Participant lives with partner	48 (48.0%)	14 (58.3%)^b^	9 (40.9%)^c^	71 (48.6%)
Duration of partnership (months), *median (IQR)*	46 (27 to 88)	45 (24 to 84)	48 (36 to 60)	48 (27 to 86)
Birthplace*, n (%)*				
South Africa	79 (79.0%)	14 (56.0%)	22 (88.0%)	115 (76.7%)
In Gauteng Province (where clinics located)	33 (33.0%)	1 (4.0%)	15 (60.0%)	49 (32.7%)
Outside Gauteng Province	46 (46.0%)	13 (52.0%)	7 (28.0%)	66 (44.0%)
Outside of South Africa	21 (21.0%)	11 (44.0%)	3 (12.0%)	35 (23.3%)
Employed*, n (%)*	31 (31.0%)	21 (84.0%)	7 (28.0%)	59 (39.3%)

IQR, interquartile range Participants at sites one and two were pregnant at the time of enrolment; at site three, participants were postpartum.

a Data on prior pregnancies not collected at site three; denominator here is 125.

b Missing 1 response at site two (n = 24).

c Missing 3 responses at site three (n = 22).

Overall, 76.7% of the participants were born in South Africa, with substantial variability between sites. Of the 23.3% of participants born outside of South Africa, most were from Zimbabwe (77.1%). Of those participants born in South Africa, more were born outside of Gauteng Province – where Johannesburg and all three clinics are located – than within, again with site variability, notably at site three. All women reported currently living Johannesburg, in communities near their respective study site.

Over one‐third of women were currently employed (36.3%), however at site two, this was as high as 84.0%. Of women currently working, the most common profession was cleaning/domestic work and median monthly salary was R3150 (USD~245 [IQR: 2450‐3900, USD~190‐300]).

### 3.2 Frequency and duration of travel

**Figure 1:**
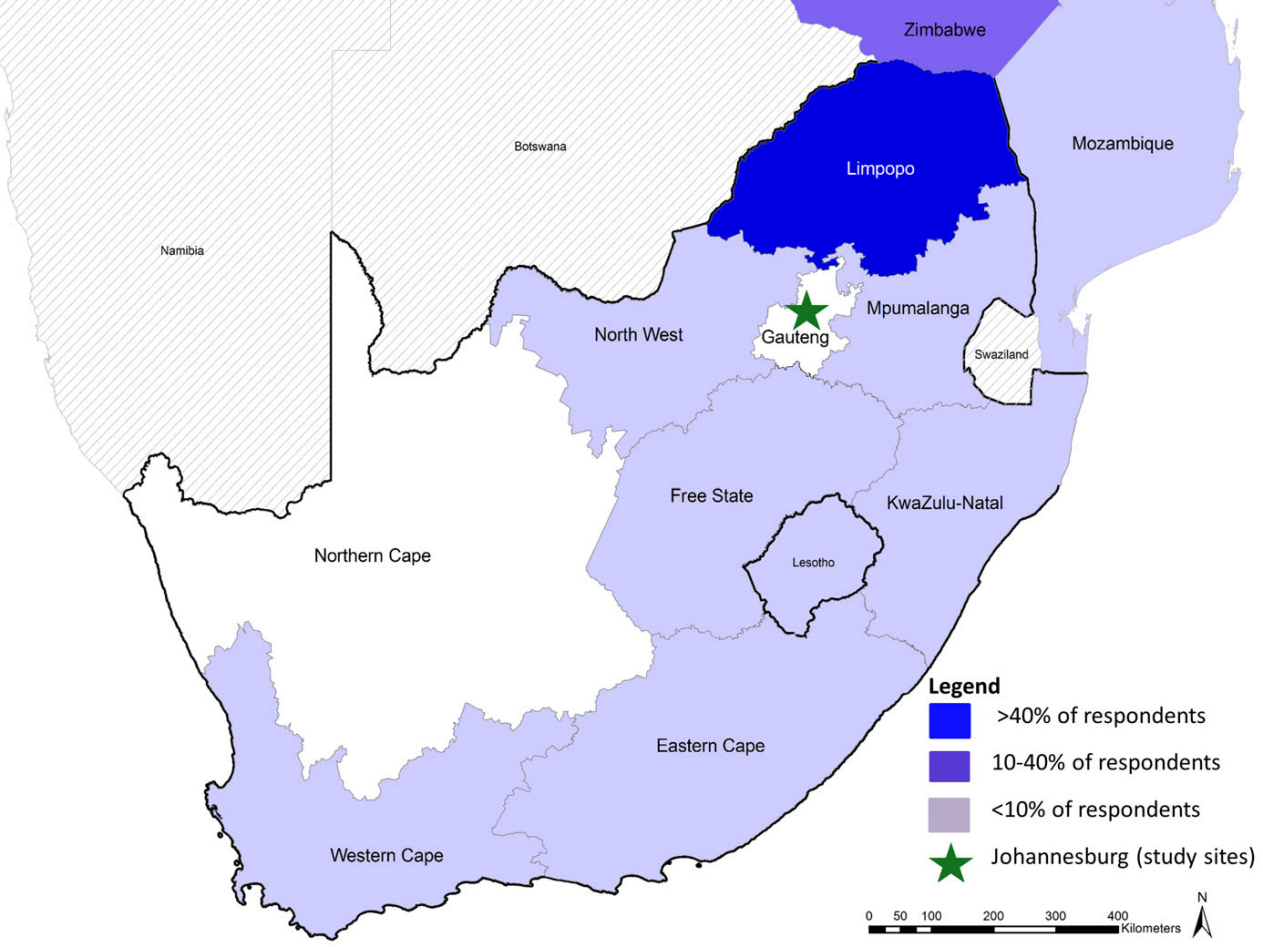
Distribution of destinations (n = 65) among the 44% of participants who reported travel around the time of delivery. Not pictured: Nigeria (1 participant; 1.5%)

Intended (site one and two) and actual (site three) travel outcomes are displayed in Table 2. At site one and two, nearly all participants planned to deliver their baby at a healthcare facility in Johannesburg; at site three, all participants gave birth at a hospital in Johannesburg. Overall, nearly half of participants reported travel around the time of delivery (44.0%); this varied from 36.0% at site one to 60.0% at sites two and three. Travel was three times more common after delivery than before. Median duration of stay during travel (before or after delivery) was 30 days (IQR: 24 to 90), but varied from 60 days at site one and two to seven days at site three. Two participants (1.3%) indicated that the move would be permanent. Location of travel is visually displayed in Figure 1. The majority of travel around delivery at all sites was to a different province in South Africa (76.9%), most frequently neighbouring Limpopo Province. Of the 18.5% of women reporting international travel, 66.7% reported travel to Zimbabwe, with Lesotho, Mozambique, and Nigeria mentioned by the remaining one‐third.

**Table 2:** Participant travel outcomes (n = 150)

** **	Site one (n = 100)	Site two (n = 25)	Site three (n = 25)	Total
Intended or actual delivery in Johannesburg, *n* (%)	92 (92.0%)	24 (96.0%)	25 (100.0%)	141 (94.0%)
Travel outside of Johannesburg before or after delivery, *n* (%)	36 (36.0%)	15 (60.0%)	15 (60.0%)	66 (44.0%)
Travel before delivery^a^	9 (9.0%)	4 (16.0%)	3 (12.0%)	16 (10.7%)
Travel after delivery^a^	27 (27.0%)	14 (56.0%)	13 (52.0%)	54 (36.0%)
Median duration of stay (days), *median (IQR)*	60 (30 to 90)	60 (21 to 120)	7 (3 to 30)	30 (24 to 90)
Travel location (among those who planned to travel)				
In Gauteng Province, but out of Johannesburg	1 (2.8%)	0^b^	2 (13.3%)	3 (4.6%)
In South Africa, but out of Gauteng Province	29 (80.6%)	11 (78.6%)^b^	10 (66.7%)	50 (76.9%)
Outside of South Africa	6 (16.7%)	3 (21.4%)^b^	3 (20.0%)	12 (18.5%)

a An individual participant could travel before and after delivery, thus responses may exceed the total here. At sites one and two, pregnant women discussed intended travel; at site three, postpartum women discussed actual travel.

b One participant at site two reported her intention to travel but did not know her location plans at the time of the interview.

### 3.3 Themes emerging from open‐ended responses

In the open‐ended responses, we identified three distinct themes: staying with family, childcare and children separated from the mother, and the plans for continuing care. Quotes selected as particularly illustrative of these themes are highlighted in Table 3. Those who were born outside of Johannesburg described a cycle of frequent travel between urban Johannesburg and a distant area considered “home.” Life in Johannesburg, as reported, involved working or looking for work; after delivery of a baby, concerns grew about childcare and security. They reported a lack of social support in the urban environment – of “no one who can look after me” – and stressed the importance of relying on extended family outside of Johannesburg for support and childcare.

**Table 3:** Illustrative quotes related to specific themes identified in open‐ended responses

Staying with family	“It's my home place. I frequently visit my family.” – Participant 5, site one
	“I will have maternity leave for four months and I will go home…just to see people.” – Participant 103, site two
	“Before I delivered yes, I would go to Pretoria…then after I delivered I went to the Free State. [I went] To see…my in‐laws in the Free State and in Pretoria was to see my husband, the father of my children. When I am in the Free State it can take a month for me being there, and then I come back. In Pretoria, I would stay about 2 or 3 months then I come back. – Participant 129, site three
	“I want my mother to look after me and the baby after delivery.” – Participant 19, site one
	“Going down to KwaZulu‐Natal [Province], because here I may not have anyone else to take care of me. At home I have my sisters. They will be able to take care of me.” – Participant 123, site two
	“This is my first child so I want my grandmother to teach me some other things about the baby.” – Participant 25, site one
	“Here there is no adult…and this is my first child; I don't know anything” – Participant 107, site two
Childcare and children separated from the mother	“My problem is that I knock off [leave work] at night. The shop closes at eight. Just imagine where the baby will be at eight?…That's why I say I don't know; I'm not sure what I'm going to do.” – Participant 118, site two
	“I think will find someone to look after my baby. A small baby cannot go to crèche [daycare] because I will be working and knocking off [leaving] at nine. I don't want to…trouble the baby. But at least the baby will be comfortable.” – Participant 112, site two
	“I will go back to work after delivery so I will leave the baby with my mother.” – Participant 88, site one
	“After four or five months, maybe I will take it [the infant] to my mother then, because I'm working…because my salary can't afford to pay crèche.” – Participant 101, site two
	“I just want to see my other child and my mother.” – Participant 15, site one
	“I want to show my mother the baby and see my other kids.” – Participant 74, site one
	“My mother and my father are in [Limpopo Province], and my second‐born child. In a year, I go there three times.” – Participant 114, site two
	“And the children, the other two, are in Zimbabwe… like April, I go to see them; December I go to see them [twice a year]… if I go there in April, I stay for one week, when I go in December, I stay for three weeks.” – Participant 105, site two
Plans for continuing care	“I will come to the [study] clinic, I'll tell them I want to travel, then they give me the medication and everything; then I make sure that the next appointment, I have to be back.” – Participant 111, site two
	“No, I did not go to the clinics in those locations; I travelled with my medication.” – Participant 133, site three
	“I will come to the [study] clinic first before I leave.” – Participant 112, site two
	“For the baby, I can still go there [outside Johannesburg]. But for me if they gave me a date [appointment], I will come on that date. I prefer to attend clinic this side [here]…It is because if I keep changing clinics and go here and there I won't get the same treatment there that I get here, so I prefer that I continue with the way I am getting treatment now.” – Participant 112, site two
	“I will have maternity leave for four months and I will go home. But I will not go home around the time to take my child or me to the clinic. They said I must bring the baby when its two weeks old and then again after ten weeks if I haven't forgotten. So I will wait to do all those things because at home I cannot do that…I am HIV positive and I know it. So they say the situation at the clinics back home is poorer than what I see here… So I can take the baby home after I have the two weeks visit; so they see it at home and then come back here and wait for the ten weeks visit.” – Participant 103, site two
	“I only took him when it was his date to get immunized, I took him to…a clinic in Free State [Province] for him to be immunized.” – Participant 129, site three
	“Yes, I did [visit a new clinic], because my baby contacted malaria…in Nigeria.” – Participant 130, site three
	“The child was taken to the grandparents in Limpopo [Province]. Yeah, no [I didn't seek care for the child]. He [the infant] was OK. He did not get any sickness or anything.” – Participant 135, site three

A prominent theme in participants’ reported experiences was travelling to stay with family. Nearly all participants who travelled during the peripartum period said they did so to visit family, most commonly their mother or mother‐in‐law. “There is no one here who can help me with the baby, so I want to be home with my mother,” one participant reported, demonstrating how mothers at “home” were portrayed as comforting, supportive, and knowledgeable about baby care. Relying on family for help with the new baby and their own postpartum recovery was the most frequent reason for staying with family. This was especially noted among first‐time mothers who described turning to mothers and grandmothers for learning. By comparison, one participant who reported family outside of Johannesburg described anticipating a different experience with her current pregnancy, her second: “I don't feel like going home. The first one I delivered at home; now I want…to do it myself so that I can learn and take responsibility.”

Childcare was a pressing concern for respondents who were employed or looking for work. Nearly all of the employed participants planned to return to work after delivering their baby, and travel was planned to coordinate with maternity leave. Even those respondents who did not plan to travel reported difficulty in coordinating working hours with childcare and paying for day care. Participants who reported travel often acknowledged leaving the infant in the care of the child's grandmother outside of Johannesburg so that the mother could return to work in Johannesburg. Similarly, respondents sometimes referred to their other children who live full‐time at the family home outside of Johannesburg. One employed participant living in an informal settlement described the relative safety of her family's home outside of Johannesburg for raising a child, stating, “It is not safe in the shack for a small baby.” Of note, no participant at site three mentioned leaving the baby at a location outside of Johannesburg.

All women expressed their plan to continue seeking healthcare in Johannesburg for themselves and their child after delivery. However, very few respondents who travelled planned to seek care at the new location. Some participants described alerting the clinic in Johannesburg in advance of their travel to ensure that they would have sufficient ART, and also timing the travel as to not interfere with upcoming clinic visits. Quotes like, “I will take the baby [home outside of Johannesburg] once I have finished everything here at the clinic,” demonstrate how some participants anticipate their travel and manage their clinic visits accordingly. Others cited concern with the quality of care at clinics outside of Johannesburg as a reason for continuing care in Johannesburg. Nearly all participants who intended to seek care elsewhere did so only for the infant, not for themselves. Some participants noted seeking care for the baby at the new location, but preferring to continue regular, adult HIV care in Johannesburg to avoid disruption. While some participants noted adhering to the newborn immunization schedule while travelling, others described only accessing care while travelling only in the event of illness.

## 4 Discussion

To our knowledge, this is the first study to explore characteristics and motivations for long‐distance travel among peripartum, HIV‐positive women in sub‐Saharan African. The impact of frequent mobility on engagement in care, particularly lifelong HIV care, is an emerging concern. Studies of migration and health usually focus on international migration, instead of circular, internal movement, which is prominent in sub‐Saharan Africa. Earlier studies have noted that mobility among peripartum women may interfere with engagement in HIV care 8, 18, 29, 30. Through this study, we sought to understand the timing and motivation of travel around delivery, and how it impacts women's choices about healthcare. We found very high frequency of internal migration and subsequent travel, particularly at two of our study sites. Travel patterns showed movement throughout South Africa, covering eight of the country's nine provinces. In addition, we also found substantial international migration, particularly to and from Zimbabwe, and most notably at site two, and short‐term travel to four countries during the peripartum period.

A strength of this study is that we report data from three different study sites in Johannesburg, and indeed, we noted substantial differences in the patient mobility at each site, reflecting the diversity of urban patient populations. Anecdotally, we had been told to expect less migration at site three, given that is an older and more well‐established community than the areas served by sites one and two, and indeed our data reflect this. Our findings are consistent with a 2013 survey within the City of Johannesburg that found 32% of respondents had moved from elsewhere in South Africa and 13% from outside of South Africa 31. At site three, far more participants were born nearby than seen at sites one and two. Travel during the peripartum period reflected this, with participants at site three less likely to report a lengthy stay outside of Johannesburg. At site three, short‐term travel often was due to attending funerals, rather than to seek help from relatives after delivery, as was the case at the other two sites. The median length of stay away for respondents at site three was only seven days.

Respondents reported their intention to return to their Johannesburg clinic to resume their HIV care after delivery, with some reporting alerting their Johannesburg clinics of their intended travel plans to ensure they would have sufficient ART. When participants did talk about visiting a clinic while travelling, it was almost exclusively for the baby, particularly to adhere to the immunization schedule. Our earlier work has shown a trend of mothers taking ART through pregnancy to protect the baby, but failing to see the importance of continued care for themselves 32. It is possible that respondents were presenting an optimistic scenario about continuing their care after delivery in order to appeal to the interviewer, a limitation of self‐reported data. We recently found evidence of women continuing HIV care elsewhere in South Africa after initiating ART in Johannesburg, 20 so women may indeed visit a clinic in their “home” area, particularly if their stay is extended. South Africa has a fragmented healthcare system, and the lack of national, networked electronic medical records means that a patient presenting at a new clinic cannot access her records from the first. Thus, the healthcare system in South Africa – and many neighbouring countries in sub‐Saharan Africa – is not equipped to adequately care for a highly mobile population.

South Africa Labour Law mandates that pregnant women are eligible for four months of unpaid maternity leave 33. While only 39% of our participants overall were employed, we found that mobility in our study very much revolved around employment. Most notably at site one and two, women moved to Johannesburg pursuing employment. At site two, 84% of women were employed, a far higher proportion than at site one and three. Still, current employment and job seeking was raised frequently in our interviews at all sites. When employed, time off work for holidays is often spent back in the other home, visiting family, including other children. This means that time off may be used for travel instead of visiting the clinic. Time in Johannesburg, according to participant accounts, focused on finding and keeping a job as a top priority.

Particularly at sites one and two, we found continued strong ties to “home” areas outside of Johannesburg. Participants noted that they often moved to Johannesburg alone and frequently identified a lack of social support in the city and safety concerns. We identified a reliance on family in distant areas, especially mothers and mothers‐in‐law for help with the baby, even going so far as to leave the baby in the care of distant family members so that the mother can return to work in Johannesburg. This was an alarming finding since all of their infants are HIV exposed. Leaving the baby behind was a frequently expressed option for both the current pregnancy, as well as for their earlier children. Even children who are HIV negative, but HIV exposed, are at increased risk of mortality compared to HIV‐unexposed children 34. Further research is needed to explore extended family members’ knowledge of HIV and caring for HIV‐exposed children, and to explore the outcomes of HIV‐exposed children raised by extended family members.

Our study has several limitations. First, all of our clinics were based in Johannesburg. While this is the largest city in South Africa, and one of the largest on the African continent, it does not necessarily reflect the mobility seen within other urban areas in South Africa. As our findings show, mobility patterns can vary substantially even within different areas of the same city. Additional research is needed to confirm our findings in different settings. Second, it is possible that women who were already travelling before delivery were omitted from the sample in site one and two. Third, the data were collected from three different studies; while this is a strength of the study, increasing population diversity and generalizability of the findings and the questions were consistent across sites, it is possible the lack of consistency in our participant population may alter our findings, distorting the generalizability of our results. Intended travel may have differed from actual travel. Reporting engagement in HIV care and ART adherence outcomes was beyond the scope of this analysis, so we are unable to report the actual loss from care among these participants. Lastly, it is possible that our sample size (n = 150) prohibited us from achieving full representation; however, it allowed for meaningful dialogue and facilitated analysis. Ultimately, we think the utility of our study is to alert HIV care providers in all plausibly similar African settings of the possibility of loss to follow‐up of postpartum women who travel after delivery and to encourage them to consider interventions that may positively impact highly mobile postpartum women.

Our finding of frequent mobility during the brief peripartum period has potentially important public health implications. While this study did not measure engagement in care, we identified frequent long‐distance travel among a population at high risk of dropping out of ART care. Continuous engagement in care is particularly important for HIV patients since HIV requires lifelong treatment with high treatment adherence; patients who stop and restart ART increase their risk of drug resistance, treatment failure and ultimately, death 35, 36, and the postpartum period is associated with increased viremia 37. Even those who remain in care but miss multiple ART appointments within the first six months of initiation are at increased risk of poor CD4 responses and failure to achieve viral suppression 38, a key consideration within the context women initiating ART during pregnancy. Our results suggest that public healthcare systems in South Africa and beyond should anticipate a mobile population, particularly around pregnancy, and adapt to improve care. An easy start is to ask a patient if she anticipates upcoming travel, discussing duration and a plan to ensure continuous care for herself and the baby, including adequate ART supply. The amount provided to patients varies depending on patient characteristics and clinic policy, but often is just 30 days. Numerous interventions to improve postpartum retention overall have drawn recent attention 39; our findings particularly support those that improve patient tracking and two‐way communication between patients and facilities.

## 5 Conclusions

In our study of HIV‐positive, pregnant and postpartum women in care in Johannesburg, South Africa, we found high levels of movement from areas within South Africa, and other countries, that largely was driven by employment aspirations. At two of our sites, we found evidence of long‐distance travel lasting a median of two months, and at all sites, a reliance upon family members for care and comfort after having a baby. In addition, we found ample evidence of women leaving their HIV‐exposed children with family members outside of Johannesburg in order to return to work. Our results highlight the frequent mobility of women in the peripartum period and help to inform our understanding of loss to follow‐up among HIV‐positive, postpartum women. Our findings support healthcare system improvements that will facilitate care across multiple facilities to adapt and accommodate a mobile population.

## Funding

This work was supported by the National Institute of Mental Health of the National Institutes of Health under Awards K01MH107256 (K. Clouse, PI) and P30MH062294 (support to S. Vermund). Additional support was provided by through a crowdfunding effort at Boston University, and the United States Agency for International Development (USAID) under Cooperative Agreement AID 674‐A‐12‐00029 (HE^2^RO staff), AID‐674‐A‐12‐00033 (Witkoppen Health and Welfare Centre). The content is solely the responsibility of the authors and does not necessarily represent the official views of the NIH, USAID, or the US Government.

## Competing interests

The authors declare no competing interests.

## Supporting information


**Table S1.** Description of three parent studies in Johannesburg, South Africa, providing data for the present analysis
**Table S2.** Mobility‐related data measurements used at three sites.
