# Supplementary material for: “I will leave the baby with my mother”: Long‐distance travel and follow‐up care among HIV‐positive pregnant and postpartum women in South Africa
Source: J Int AIDS Soc. 2018 Jul 19;21(Suppl Suppl 4):e25121. doi: 10.1002/jia2.25121 (PMC6053484; doi:10.1002/jia2.25121)
Supplement: Supplementary file 1 — Table S1. Description of three parent studies in Johannesburg, South Africa, providing data for the present analysis Table S2. Mobility‐related data measurements used at three sites. [file JIA2-21-e25121-s001.docx]

**Supplementary Table 1. Description of three parent studies in Johannesburg, South Africa, providing data for the present analysis**

|  | **Site one** | **Site two** | **Site three** |
| --- | --- | --- | --- |
| **Study objective** | To determine the feasibility of an economic incentive intervention aimed at improving retention in postpartum HIV care | To identify the drivers and characteristics of population mobility among HIV-positive pregnant and postpartum women in South Africa | To characterize health service access, navigation and utilization among postpartum women co-infected with HIV and a metabolic disorder. This was a sub-study of a larger study – the Soweto First 1000 Days Cohort – a cohort of women recruited during antenatal care and followed longitudinally to examine maternal factors and their impact on fetal and infant growth and development |
| **Sample size** | 100 | 25 | 25 |
| **Study design** | Prospective cohort | Prospective cohort | Cross-sectional sub-study |
| **Enrollment dates** | May 2015 – March 2016 | October 2016 – April 2017 | August – December 2016 |
| **Eligibility criteria** | - Adult (≥18 years) - HIV-positive - Pregnant - Able to speak and understand English | - Adult (≥18 years) - HIV-positive - Pregnant | - Adult (≥18 years) - HIV-positive - Recently postpartum (6-18 months prior to enrollment) - Co-infected with a metabolic disorder |
| **Study recruitment methods** | Approached consecutively during routine antenatal care | Approached consecutively during routine antenatal care | Approached consecutively during follow-up care |
| **Data collection methods** | One-time questionnaire at enrollment, using categorical options with the possibility of open-ended responses | In-depth interview at enrollment | One-time in-depth interview |

**Supplemental Table 2. Mobility-related data measurements used at three sites.**

| **Indicator** | **Site one** | **Site two** | **Site three** |
| --- | --- | --- | --- |
| **Birth location** | Where do you plan to deliver your baby? *[tick one]* 1. In Gauteng *[specify hospital]*  2. In South Africa, but not Gauteng *[specify province]*  3. Outside of South Africa *[specify country]* | Where do you plan to deliver your baby? | Where did you deliver your baby? |
| **Travel before/after delivery** | Do you plan to travel to stay with family or friends who live outside of Johannesburg after the baby is born?  Yes/ No *[If Yes]:* Where do you plan to go?  1. In Gauteng, but not Johannesburg [specify city/township]  2. In South Africa, but not Gauteng [specify province and city/township]  3. Outside of South Africa [specify country]  Who will you stay with there? *[tick all that apply]*  1. My boyfriend or husband  2. A family member [specify relationship]  3. A friend who is not my boyfriend or my family   How long do you plan to stay?  \|__\|\|__\| days *or tick \|__\| if permanent move*   What is the purpose of your trip? *[open ended]* | Do you plan to travel outside Johannesburg before or after you give birth? When do you plan to travel? How long will you stay? Why will you go? | Did you travel outside Johannesburg before or after you give birth? Why?  How long did you stay?  Why did you go? |
| **Seeking care in new area** | Do you plan to receive care for you and your baby at a clinic in the new area?  Yes/No/I'm not sure *[If Yes]* Do you know the name of the clinic? *[record clinic name and province]* | Do you plan to seek care for you and/or your baby at the new location? Where? | Did you seek care for you and/or your baby at the new location? Where? |
